# A novel *DLG4* variant causes *DLG4*-related synaptopathy with intellectual regression

**DOI:** 10.1038/s41439-023-00260-x

**Published:** 2024-01-05

**Authors:** Sachi Tokunaga, Hideki Shimomura, Naoko Taniguchi, Kumiko Yanagi, Tadashi Kaname, Nobuhiko Okamoto, Yasuhiro Takeshima

**Affiliations:** 1 Department of Pediatrics, Hyogo Medical University School of Medicine, Nishinomiya, Hyogo Japan; 2 Department of Genome Medicine, National Center for Child Health and Development, Tokyo, Japan; 3 Department of Medical Genetics, Osaka Women’s and Children’s Hospital, Osaka, Japan

**Keywords:** Genetic testing, Autism spectrum disorders

## Abstract

*DLG4*-related synaptopathy is a neurodevelopmental disorder caused by a *DLG4* variant. We identified a novel de novo heterozygous frameshift variant, NM_001321075.3(*DLG4*):c.554_563del, in a Japanese girl. Intellectual regression without motor delay was observed at 2 years of age, and she was diagnosed with autism spectrum disorder and attention-deficit/hyperactivity disorder. Recognizing the possibility of *DLG4*-related synaptopathy in patients with intellectual regression is important for ensuring an accurate diagnosis.

Discs large MAGUK scaffold protein 4 (*DLG4)* encodes postsynaptic density protein 95 (PSD-95), which is expressed in various tissues, including the brain. Haploinsufficiency of *DLG4* likely impairs PSD-95 activity and disrupts synaptic function during critical developmental periods. This alters the synaptic plasticity needed for functional adaptation and regulation of learning and behavioral processes, leading to neurodevelopmental disorders in these individuals^1,2^. Rodriguez-Palmero et al. designated this group of disorders as *DLG4*-related synaptopathy^2^. Approximately 0.05% of individuals with intellectual disabilities (IDs) may have variants in *DLG4*^2,3^. Some *DLG4* variants have been identified during screening for new candidate genes for ID^4–6^, developmental disorders^7^, schizophrenia, autism spectrum disorder (ASD)^8^, and cerebral visual impairment^9^. *DLG4*-related synaptopathy can cause various symptoms. Approximately 98% of patients present with ID and motor delay, and one-third exhibit intellectual regression. However, few patients display intellectual regression without motor delay.

In this study, we report the case of a 5-year-old girl who presented with intellectual regression and was found to have a novel de novo frameshift variant of the *DLG4* gene.

The girl presented to our hospital with intellectual regression that developed at the age of 2 years. The patient was born via normal delivery following an uncomplicated, full-term pregnancy. She showed normal development up to the age of 2 years. She gained head control at 4 months of age, rolled over at 5 months, crawled at 8 months, and walked without support at 14 months. She could speak two-word phrases at 18 months of age, and she demonstrated social referencing. She passed her 18-month follow-up without any complications. When she turned 2 years old, her parents noticed intellectual regression, diminished eye contact, and restlessness. Occasionally, stereotypies movements were observed in her upper extremities. The patient was unable to speak meaningful words. At this time, she was diagnosed with ASD and attention-deficit/hyperactivity disorder (ADHD). She began receiving rehabilitation for the verbal delay. Her developmental quotients (DQs) were 46, 33, and 36 at 2 years 3 months, 3 years, and 4 years of age, respectively, as evaluated using the Kyoto Scale of Psychological Development.

At 5 years of age, her motor function was normal at the initial examination. Her blood test and urinalysis results, including thyroid function and metabolic screening (lactate, amino acids, and urine organic acids), were normal. Brain magnetic resonance imaging (MRI) and electroencephalography (EEG) results were also normal. Conventional chromosomal G-banding analysis showed a normal female karyotype (46, XX). Chromosomal microarray analysis revealed no detectable pathologic copy number variants. Therefore, trio-whole exome sequencing (trio-WES) was performed using the Human All Exon V6 Kit (Agilent Technologies, CA, USA) and NovaSeq 6000 (Illumina, CA, USA) with 150-bp paired-end reads. The obtained reads were aligned to GRCh38 and annotated using CompStor NOVOS and CompStor Insight (OmniTier, CA, USA). The filtering process for the candidate variants was as follows. First, variants with allele frequencies >0.01 in gnomAD, 14KJPN (jMorp) and our inhouse exome variant database were removed. Then, the remaining variants were filtered according to the assumed modes of inheritance, such as autosomal dominant, autosomal recessive, X-linked, and compound heterozygous inheritance. Finally, the trio-WES and filtering analyses identified a de novo frameshift variant of the *DLG4* gene, NM_001321075.3:c.554_563del:p.(Gly185AlafsTer4), in the patient. This variant was confirmed by Sanger sequencing (Fig. 1). This frameshift variant was not registered in public genome databases such as gnomAD and 14KJPN. A program that predicts variant effects, Mutation Taster, predicted it to be a disease-causing variant. The probability of loss-of-function intolerance score of the *DLG4* gene is 1 (pLI = 1), and frameshift variants are registered as pathogenic in ClinVar. The variant was classified as pathogenic according to the American College of Medical Genetics and Genomics guidelines (PVS1, PS2, PP3). We suspected Rett syndrome based on the intellectual regression and stereotypies movement. However, trio-WES analysis showed no pathogenic variants in the methyl-CpG-binding protein 2 (*MECP2*) gene in the patient.

**Fig. 1:**
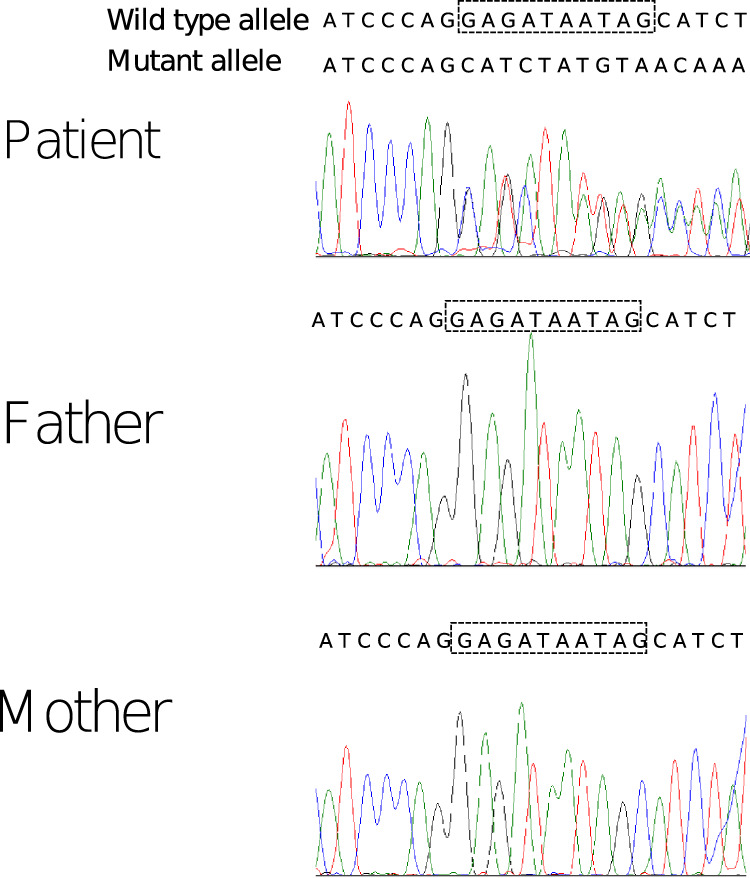
Sanger sequencing of the patient and her biological parents. The top sequence of the patient is a wild-type allele, and the bottom sequence of the patient is a mutant allele. The sequence enclosed by dotted lines was deleted in the mutant allele.

In the present case, we detected a novel heterozygous frameshift variant in *DLG4* using WES to clarify the cause of intellectual regression. Due to advancements in genetic testing technologies, approximately half of children with moderate-to-severe ID receive specific genetic diagnoses^10^. *DLG4* was one of the candidate genes found in 2104 patient–parent trios analyzed to identify candidate genes for ID^5^. In one study, 15 individual *DLG4* variants were identified in 31,058 patients with developmental disorders^3^. Diagnosing a specific ID or developmental disorder offers various benefits to patients and their families. For example, patients and families can receive information on the prognosis or expected clinical course, avoid unnecessary and redundant diagnostic tests, and discuss recurrence risks in their offspring^11^. However, it may also identify variants unrelated to these characteristics^12^. Therefore, the interpretation of these results and their explanation to patients remains challenging^12^.

Since 53 affected individuals have been reported^2^, the opportunities to explore genotype–phenotype correlations are gradually expanding. *DLG4*-related synaptopathy has been reported to cause mild-to-severe ID. Frameshift and nonsense variants similar to those in our case with severe ID have been reported. However, these cases did not show regression and developed epilepsy^2^. The type of epilepsy varies and includes generalized and partial seizures. Currently, identifying genotype–phenotype correlations or functional hotspots is challenging. It is necessary to accumulate more case studies to gain a better understanding of the genotype–phenotype correlations in *DLG4*-related synaptopathy.

In the present case, the patient presented with intellectual regression, ASD, and ADHD. Rodriguez-Palmero et al. reported the clinical and genetic features of 53 patients with *DLG4* variants^2^. *DLG4*-related synaptopathy manifests as developmental delay, muscular hypotonia, ID, ASD, ADHD, abnormal movement (stereotypies, ataxia, and dystonia), epilepsy, ophthalmologic abnormalities, and Marfanoid habitus. Marfanoid habitus manifests as ID and skeletal signs suggestive of Marfan syndrome (OMIM 154700) but does not meet international criteria, such as aortic root dilatation and lens dislocation^6,13^. The most frequent clinical feature of *DLG4*-related synaptopathy is ID with motor delay. A study assessing 53 patients revealed that only one of them had normal motor development. In addition, approximately half of the patients developed epilepsy, and in some cases, seizures developed more than 10 years after onset^2^. Since our patient has not developed motor delay or epilepsy, careful observation is needed for the early detection of these symptoms.

The most specific feature in this case was intellectual regression without motor delay. The patient showed normal development since birth, but she presented with intellectual regression at the age of 2 years. We attempted to differentiate between the various diseases that could cause intellectual regression. We differentiated between metabolic and degenerative disorders using blood tests, urinalysis, and brain MRI. We suspected Landau–Kleffner syndrome because of the loss of language function; however, the EEG results were normal. We also suspected Rett syndrome; however, *MECP2* was also normal. Finally, the patient was diagnosed with *DLG4*-related synaptopathy using WES. Rodriguez-Palmero et al. reported that approximately one-third of patients show developmental regression^2^. *DLG4*-related synaptopathy may cause nonspecific symptoms. In the differential diagnosis of intellectual regression, *DLG4*-related synaptopathy should be considered.

The average age of onset of regression in individuals with *DLG4*-related synaptopathy was 4 years, while the onset of epilepsy was 6 years^2^. Individuals with epilepsy are more likely to experience regression in motor development and/or language skills than those without epilepsy^2^. EEG abnormalities, including electrical status epilepticus during slow-wave sleep and hypsarrhythmia, have also been reported.

PSD-95, encoded by the *DLG4* gene, plays a crucial role in synaptic maturation, dendritic morphology, and the regulation of the function of the glutamate receptors by N-methyl-d-aspartic acid (NMDA) and α-amino-3-hydroxy-5-methyl-4-isoxazolepropionic acid (AMPA)^1^. When PSD-95 was removed from neurons, there was a significant reduction in synaptic transmission mediated by AMPARs and NMDARs^14^. Regression can occur due to the dysfunction of these excitatory neurons.

In conclusion, we report the case of a 5-year-old girl who presented with intellectual regression and was found to have a novel frameshift variant in *DLG4*. In patients presenting intellectual regression, *DLG4*-related synaptopathy should be considered for appropriate diagnosis.

This study was approved by the Central Ethics Committee at Tohoku University on January 26, 2021 (approval number 20851). We obtained written informed consent from the patient’s parents for the publication of this case report and genetic analysis.

## HGV Database

The relevant data from this Data Report are hosted at the Human Genome Variation Database at 10.6084/m9.figshare.hgv.3351.
